# Postmortem genetic diagnosis of hereditary leiomyomatosis and renal cell carcinoma syndrome: Identification through normal kidney tissues after surgical removal

**DOI:** 10.1002/iju5.12820

**Published:** 2024-12-26

**Authors:** Shodai Suzuki, Yoshiyuki Yamamoto, Taigo Kato, Koji Hatano, Takahiro Matsui, Kae Hashimoto, Takako Miyamura, Yoji Nagashima, Norio Nonomura, Atsunari Kawashima

**Affiliations:** ^1^ Department of Urology Osaka University Graduate School of Medicine Suita Osaka Japan; ^2^ Department of Pathology Osaka University Graduate School of Medicine Suita Osaka Japan; ^3^ Department of Genetic Counseling Osaka University Graduate School of Medicine Suita Osaka Japan; ^4^ Department of Pediatrics Osaka University Graduate School of Medicine Suita Osaka Japan; ^5^ Department of Surgical Pathology Tokyo Women's Medical University Hospital Tokyo Japan

**Keywords:** cabozantinib, comprehensive genome profiling, FH, hereditary leiomyomatosis and renal cell cancer, renal cell carcinoma

## Abstract

**Introduction:**

Hereditary leiomyomatosis and renal cell cancer is an autosomal dominant disorder caused by germline mutations in the *FH* gene and is associated with poor prognosis of aggressive renal cancer.

**Case presentation:**

A 33‐year‐old man presented with asymptomatic gross hematuria and was diagnosed with a right renal tumor, cT3aN1M0. He underwent open radical nephrectomy, and pathological examination revealed papillary renal cell carcinoma. Despite aggressive treatment, the disease progressed rapidly, and discussions regarding genetic testing could not take place during his lifetime, although circulating‐tumor DNA showed mutation of *FH* gene. After death, his wife requested postmortem genetic testing. Genetic analysis using DNA extracted from normal kidney tissues in surgical specimens (blood sample absence) confirmed the *FH* mutation, and hereditary leiomyomatosis and renal cell cancer was diagnosed posthumously.

**Conclusion:**

This highlights the utility of postmortem genetic testing of surgical specimens to diagnose hereditary leiomyomatosis and renal cell cancer and provide genetic counseling to families, despite limitations during the patient's life.

**Abbreviations & Acronyms**
2SC: *S*‐(2‐succino)‐cysteine
Ave: avelumab
Axi: axitinib
BSC: best supportive care
Ca: calcium
Cabo: cabozantinib
CGP: comprehensive genome profiling
CK: cytokeratin
CT: computed tomography
F1LCDx: FoundationOne® Liquid Companion Diagnostics
FH: fumarate hydratase
FHD‐RCC: fumarate hydratase‐deficient renal cell carcinoma
HLRCC: hereditary leiomyomatosis and renal cell carcinoma syndrome
ICI: immune checkpoint inhibitors
IMDC: International Metastatic Renal Cell Carcinoma Database Consortium
LN: lymph node
MRI: magnetic resonance imaging
Nivo: nivolumab
ORR: objective response rate
PD: progressive disease
PeD: peritoneal dissemination
PR: partial response
RCC: renal cell carcinoma

**Keynote message**

Herein, we report a case of HLRCC diagnosed using postmortem genetic testing. Testing of circulating‐tumor DNA during life revealed the possibility of HLRCC, which was diagnosed based on normal kidney‐derived DNA from a surgical specimen.

## Introduction

FHD‐RCC is a rare subtype of RCC, which is associated with HLRCC syndrome.^1^, ^2^ HLRCC is an autosomal dominant disease, and the causative gene is *FH*.^1^ This syndrome is characterized by loss of *FH* heterozygosity, leading to loss of FH protein expression and increase in 2SC in tumors.^3^ Cutaneous leiomyomas, uterine fibroids, and RCC develop in over 70%, 80%, and 15%–20% of patients with pathogenic variants in *FH*, respectively.^4^, ^5^, ^6^ The prognosis for FHD‐RCC is generally poor, with high local invasiveness and metastasis.^3^, ^7^, ^8^ HLRCC diagnosis requires the identification of a heterozygous variant of *FH* in the germline.^9^ Here, we report a case in which HLRCC was diagnosed using a normal kidney from surgical specimen after the patient's death, and appropriate genetic counseling for his family was provided.

## Case presentation

**Fig. 1:**
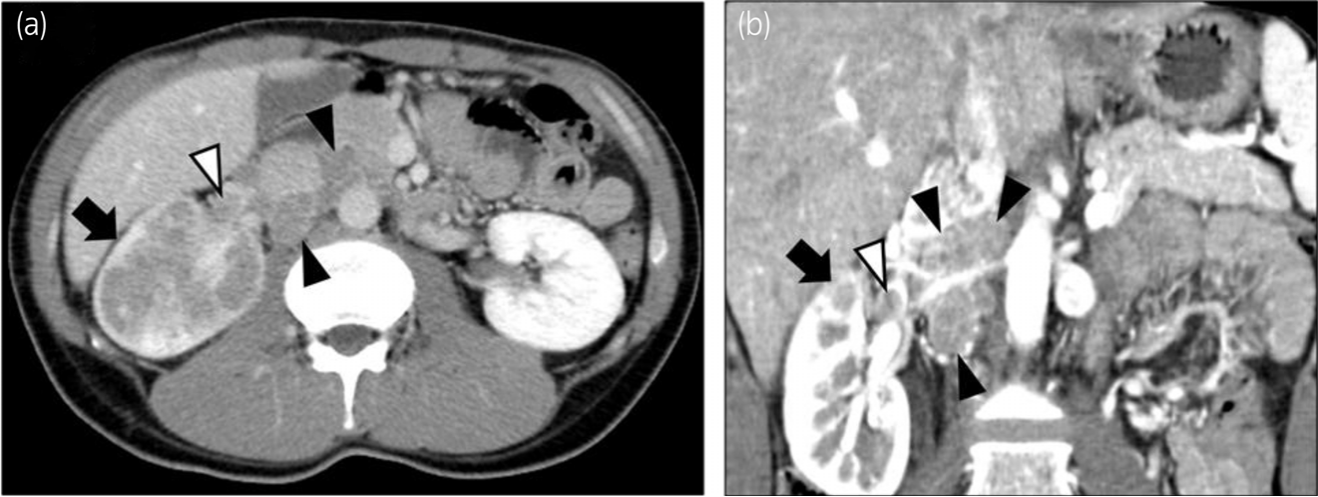
Abdominal contrast CT shows a right renal tumor (Black arrow) of 66 mm in size with thrombus (White arrow heads) in right renal vein and multiple enlarged LNs (Black arrow heads). (a) Axial image, (b) Coronal image.

A 33‐year‐old man presented with asymptomatic gross hematuria. CT tomography revealed a right renal tumor and multiple enlarged LNs (Fig. 1). The patient was diagnosed with right renal cancer, cT3aN1M0, and underwent open radical nephrectomy and retroperitoneal LN dissection.

**Fig. 2:**
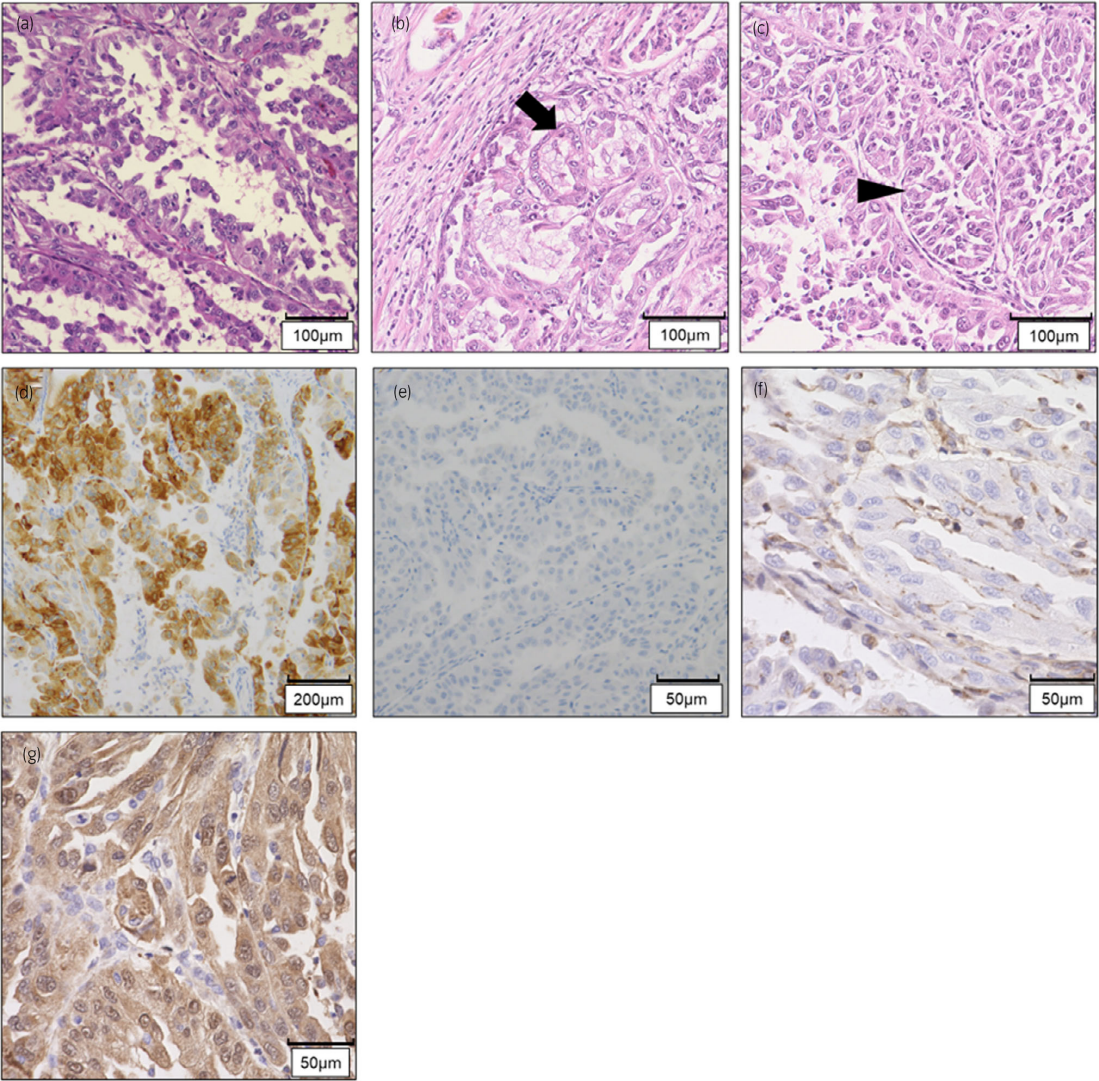
Histopathological findings of the specimen of right nephrectomy. (a–c) Hematoxylin–eosin stain (H&E); papillary structure (a), cribriform (arrow) (b), and tubular (arrow head) (c), respectively. (d–g) Immunohistochemistry examination; α‐methyl acyl‐CoA racemase (d), CK 7 (e), FH (f), and 2SC (g).

On pathological examination, the tumor had a variety of papillary, cribriform, and tubular structures (Fig. 2a–c). Immunohistochemically, the tumor cells were positive for α‐methyl acyl‐CoA racemase and negative for CK 7 (Fig. 2d,e). LN metastases were positive, and the pathological diagnosis was papillary RCC, pT3apN1.

**Fig. 3:**
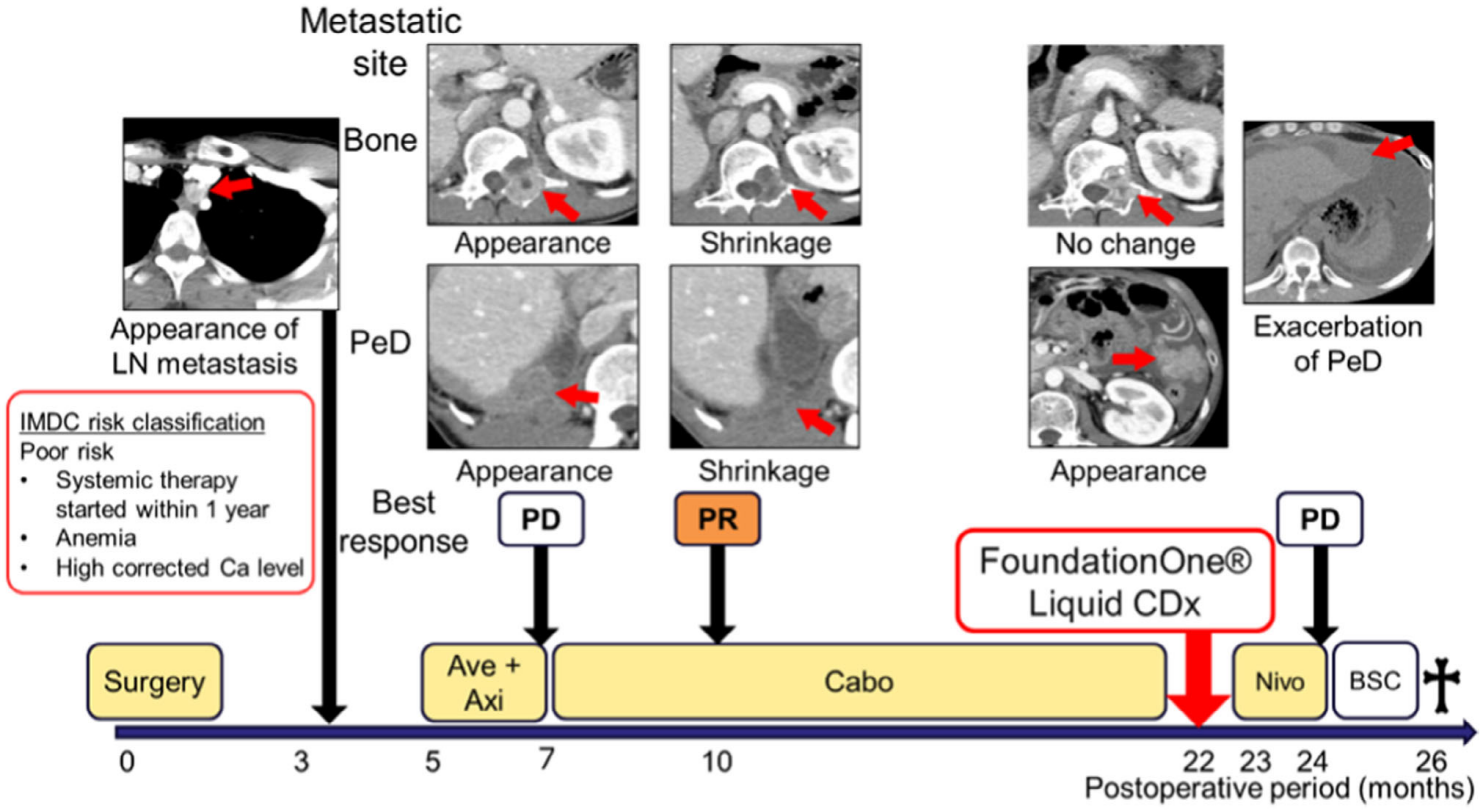
Clinical course after surgery. Arrows in CT images showed metastatic site. With the three factors: “<1 year from time of diagnosis to systemic therapy,” “anemia; 13.5 g/dL (Facility Standards, ≥13.8 g/dL),” and “high level of corrected Ca; 10.7 mg/dL,” the IMDC risk classification was determined to be poor risk. †, death.

Three months after surgery, metastatic disease was detected in the mediastinal LNs (Fig. 3). IMDC risk classification was determined to be poor risk. Ave and Axi therapy was initiated 5 months after surgery, yet bone, liver, and peritoneal metastases appeared 2 months later. Cabo was started as second‐line therapy. Three months after treatment initiation, the response was judged to be partial, and Cabo was administered for 15 months. At 22 months postoperatively, the disease progressed, and CGP using F1LCDx was performed. We chose liquid biopsy because we wanted to detect any mutations that might have arisen after drug treatment, yet no mutations eligible for treatment were identified. A secondary finding by expert panel identified only a mutation in *FH* (F57fs*4) with a variant allele frequency of 53.7%; this mutation has not been submitted to public databases, including Clinvar^10^ and gnomAD.^11^ A germline mutation was suspected; however, the disease worsened, and further testing could not be performed. Nivo was started as third‐line therapy, yet the disease progressed within a month, and he died at 26 months postoperatively.

**Fig. 4:**
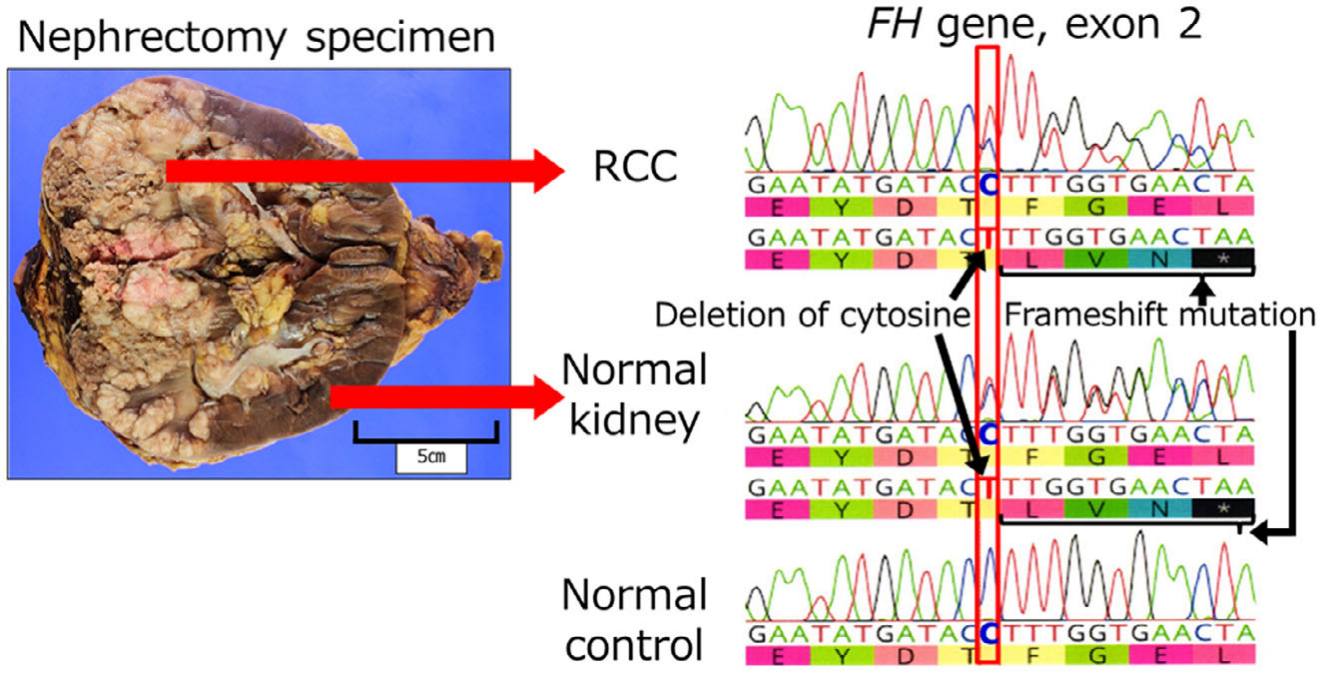
Single‐site test using nephrectomy specimens. Using DNA from normal and tumor of kidney, we performed a single‐site test, which showed the deletion of cytosine and the same *FH* mutation as that of F1LCDx.

After his death, the wife requested a genetic examination because they had two daughters aged 8 and 10 years. The family history contained no special note. His blood sample was not stored; therefore, we devised the idea of using a normal kidney sample removed during the surgery. We extracted DNA from normal and neoplastic kidney tissues and performed a single‐site test, which revealed the same *FH* mutation as that of F1LCDx (Fig. 4). Hence, the patient was diagnosed with HLRCC. Additional immunohistochemical examination showed that tumor cells were negative for FH and positive for 2SC (Fig. 2f,g), and the final pathological diagnosis was FHD‐RCC.

After genetic counseling with his wife, we made the following decisions: Genetic testing of the children was not performed because of their emotional stress at that time. When they reach 18 years of age, we are planning to ask the wife again if she would like genetic counseling for the children. Surveillance for cutaneous leiomyomatosis resulted in a dermatological consultation to check if the patient's children had skin nodules. Routine surveillance was not performed for the uterine fibroids because the disease is benign. Regarding RCC surveillance, our policy was to begin with annual ultrasound examinations around the age of 15 years.

## Discussion

FHD‐RCC is a rare and aggressive form of RCC associated with HLRCC. The median age of onset for FHD‐RCC is 40–46 years.^7^, ^12^, ^13^ Approximately 50% of patients with FHD‐RCC show metastases at initial diagnosis.^7^, ^13^ Drug therapy for metastatic FHD‐RCC has not been established. The ORR of Cabo for FHD‐RCC was 50%, with a median response duration of 14.0 months.^3^ In contrast, ORR for ICI and combination therapy was 18%.^3^ In another report, all five patients treated with Cabo plus Nivo for FHD‐RCC exhibited objective response.^14^ In this case, ICI combination therapy was ineffective, whereas Cabo monotherapy was relatively successful.

Regarding postoperative course, at the time of surgery, adjuvant immunotherapy with pembrolizumab^15^ was not available. A tissue biopsy for LN enlargement after surgery was initially considered; however, due to the difficult access, it was deemed unfeasible. Consequently, it took 2 months from the recurrence to the initiation of treatment.

F1LCDx using circulating‐tumor DNA revealed a possible germline *FH* mutation; however, because of patient's deteriorating condition, single‐site testing could not be performed while he was alive. Nephrectomy specimens contained normal kidneys, which were tested for germline mutations, and the same mutations as those in CGP were identified. Interestingly, to our knowledge, no reports exist of HLRCC diagnosed posthumously. Another group reported that postmortem identification of germline mutations from tissue DNA in various cancer has enabled appropriate surveillance and avoidance of unnecessary examinations.^16^ As CGP testing for tumors becomes more widespread, the number of cases requiring genetic testing for diagnosing hereditary conditions is expected to increase.

On surveillance, the guideline specifies that screening should generally begin 10 years before the earliest age of diagnosis onset in family members suspected of hereditary kidney cancer.^17^ GeneReviews® summarized that surveillance for RCC in patients with pathogenic variants in *FH* should include annual contrast‐enhanced MRI or CT beginning at 8 years old.^18^ Yet, the chance of developing RCC in patients with pathogenic variants in *FH* under the age of 20 years is 1%–2%.^6^ Whether their daughters have *FH* mutation remains unknown. We decided to start surveillance for RCC around the age of 15 years, initially by ultrasonography, because of radiation exposure associated with CT and financial problems associated with MRI scans.

It was reported that 5.2% of patients with RCC under 46 years had HLRCC.^19^ For patients with FHD‐RCC, the pathological diagnosis was previously considered typically papillary RCC, yet previous reports showed that only 50%–74% have papillary RCC, and some have a tubular cystic renal cell, ductal, medullary or unclassifiable carcinoma types.^7^, ^12^, ^20^ Considering that CGP is becoming increasingly popular, an increase in the number of HLRCC diagnoses is expected in the future.

In conclusion, we report a case of HLRCC diagnosed using CGP. Surgical specimens were used to perform single‐site testing for postmortem genetic diagnosis. We believe that promoting awareness of HLRCC and linking it to appropriate diagnosis and genetic counseling is important.

## Author contributions

Shodai Suzuki: Conceptualization; data curation; investigation; project administration; writing – original draft. Yoshiyuki Yamamoto: Conceptualization; investigation; project administration; writing – original draft. Taigo Kato: Supervision; writing – review and editing. Koji Hatano: Supervision; writing – review and editing. Takahiro Matsui: Data curation; supervision; writing – review and editing. Kae Hashimoto: Investigation; supervision; writing – review and editing. Takako Miyamura: Investigation; writing – review and editing; supervision. Yoji Nagashima: Data curation; supervision; writing – review and editing. Norio Nonomura: Supervision; writing – review and editing. Atsunari Kawashima: Conceptualization; project administration; supervision.

## Conflict of interest

The authors declare no conflict of interest.

## Approval of the research protocol by an Institutional Reviewer Board

It is approved by Ethics Committee in Osaka university hospital to perform genetic testing after the death of the individual, without the consent of the individual and only with the consent of the family (Approval number: 358‐3).

## Informed consent

Written informed consent was obtained from the patient for the publication of this case report and accompanying images.

## Registry and the Registration No. of the study/trial

Not applicable.

## Data Availability

Not applicable.
